# Anticholinergic Burden in Older Adults Referred to Old Age Psychiatric Liaison: A Quality Improvement Project

**DOI:** 10.1192/bjo.2025.10083

**Published:** 2025-06-20

**Authors:** Charlotte Blackmore, Jennifer Parker, Seona Duroux

**Affiliations:** University Hospitals Bristol and Weston Foundation Trust, Bristol, United Kingdom

## Abstract

**Aims:** This quality improvement project (QIP) aims to evaluate the assessment of anticholinergic burden (ACB) of medications, using a validated tool, in patients admitted to Bristol Royal Infirmary and referred to Later Life Liaison Psychiatry, aiming to increase awareness and reduce ACB where appropriate.

**Methods:** The Anticholinergic burden Effect on Cognition (AEC) validated tool was selected to assess ACB. Baseline data was collected and anonymised from 20 patients via team assessments in patient records. Data included the AEC score, medications involved, prescription indication, whether ACB was considered, and if AEC score was documented.

An educational intervention involved teaching liaison psychiatry staff on ACB, AEC and strategies for deprescribing or switching medication. The team’s knowledge was evaluated before and after teaching using questionnaires. An educational poster was displayed around the office.

Post-intervention data was collected from five additional patients, and the results were analysed.

**Results:** Baseline data showed 25% of patients (n=20) scored AEC ≥3. 30% were on multiple medications with an AEC score, 50% were prescribed antidepressants, predominantly mirtazapine and sertraline (both AEC=1). Only 15% of the assessments had a documented AEC.

Prior to the educational intervention, 71% of the team reported their ACB knowledge level as “very poor”, “poor”, or “average”. After the teaching, 71% of the team rated their knowledge as “very good”, indicating significant improvement.

Following the intervention, no patients (n=5) scored AEC ≥3, and 60% of assessments documented the AEC score.

**Conclusion:** The most prescribed medications contributing to ACB were, in order, cyclizine, mirtazapine and sertraline, aligning with current national literature. Most patients with AEC ≥3 were taking multiple drugs, leading to a cumulative effect. Of the assessments that did not document the AEC score after teaching, all had scores of 0, suggesting staff may not view this score as significant.

All psychiatry liaison colleagues acknowledged the importance of ACB, but had a knowledge gap prior to the educational intervention, which showed improvement following a well-received teaching session.

This QIP demonstrates patients interfacing with old age psychiatry liaison can have a high ACB. The liaison team are well-placed to acknowledge and review these medications collaboratively with medical colleagues. An education intervention shows improvements in assessing ACB in our service.

For sustainability, further service level interventions have been implemented, including bookmarking the AEC calculator on staff computers (medichec.com) and adding a prompt to the team’s initial assessment template to check AEC. These measures aim to continue improving patient outcomes.

